# Risk factors for lenvatinib‐induced palmar‐plantar erythrodysesthesia syndrome in patients with hepatocellular carcinoma: A retrospective study

**DOI:** 10.1002/cam4.70065

**Published:** 2024-08-27

**Authors:** Shusuke Uekusa, Maho Nemoto, Yuki Hanai, Misaki Nakashin, Sachiko Yanagino, Yoshiki Arita, Takahiro Matsumoto, Noritaka Wakui, Hidenari Nagai, Koji Higai, Kazuhiro Matsuo

**Affiliations:** ^1^ Department of Clinical Pharmacy, Faculty of Pharmaceutical Sciences Toho University Funabashi Japan; ^2^ Toho University Ohashi Medical Center Meguro‐ku Japan; ^3^ Toho University Omori Medical Center Ota‐ku Japan; ^4^ Division of Gastroenterology and Hepatology, Department of Internal Medicine (Omori), School of Medicine, Faculty of Medicine Toho University Ota‐ku Japan; ^5^ Laboratory of Medical Biochemistry, Faculty of Pharmaceutical Sciences Toho University Funabashi Japan

**Keywords:** alkaline phosphatase, hepatocellular carcinoma, lenvatinib, monocyte, γ‐Glutamyl transpeptidase

## Abstract

**Aim:**

Lenvatinib mesylate (LEN) is an oral tyrosine kinase inhibitor used to treat various cancers, including hepatocellular carcinoma (HCC). HCC treatment with LEN is associated with a very high incidence of adverse events. This study was aimed at investigating the incidence of LEN‐induced palmar‐planter erythrodysesthesia syndrome (PPES) and its relationship with patient demographics by analyzing clinical laboratory data of LEN‐treated patients with HCC.

**Methods:**

This was a single‐centre, retrospective study of patients with HCC who received LEN between April 19, 2018, and September 30, 2020. The observation period was from 1 week before the start of LEN administration to 1 month after the end of administration.

**Results:**

Overall, 75 patients with HCC were enrolled. LEN‐induced PPES was found in 48.0% (36/75 patients). In these patients, alkaline phosphatase (ALP), γ‐Glutamyl transpeptidase (γ‐GTP) and monocytes (MONO) were significantly high (ALP: *p* = 1.32 × 10^−3^, γ‐GTP: *p* = 4.25 × 10^−3^ and MONO: *p* = 0.013). The cut off values of ALP, γ‐GTP and MONO for LEN‐induced PPES were estimated at 573 U/L, 89 U/L, and 310 counts/μL, respectively. In the multivariate analysis, γ‐GTP and MONO were independent risk factors for LEN‐induced PPES.

**Conclusions:**

High γ‐GTP and high MONO were risk factors for LEN‐induced PPES.

## 1 INTRODUCTION AND BACKGROUND

HCC is the most prevalent primary liver cancer worldwide.^1^, ^2^ The global incidence of HCC has increased over the last three decades, and this trend is expected to continue until 2030.^3^ Therefore, the development of safe and effective treatments is important. Lenvatinib mesylate (LEN) is an orally administered tyrosine kinase inhibitor (TKI). LEN was first approved for unresectable thyroid cancer and was later investigated as a first‐line treatment for hepatocellular carcinoma (HCC) in a randomized Phase 3 clinical trial (the REFLECT trial) in direct comparison with sorafenib, the first drug demonstrated to improve survival in patients with advanced HCC.^4^ The American Association for the Study of Liver Diseases guidelines recommend systemic therapy with atezolizumab plus bevacizumab or durvalumab plus tremelimumab as the preferred first‐line therapy; otherwise, treatment with sorafenib or LEN is recommended.^5^

Nonetheless, LEN therapy for HCC is associated with a high incidence of adverse events (AEs). In the REFLECT trial, 94% of all patients^4^ and 100% of Japanese patients experienced treatment‐related AEs.^6^ Hypertension, hypothyroidism, and palmar‐plantar erythrodysesthesia syndrome (PPES) are characteristic AEs of LEN. We recently reported that low eosinophil counts and non‐current smoking status are risk factors for LEN‐induced hypothyroidism.^7^ In a subgroup analysis of the REFLECT study, a higher incidence of PPES was reported in the Japanese population (any grade: Japanese, 51.9%; overall, 27%; grade ≥3: Japanese, 7.4%; overall, 3%), with PPES being the most common AE.^4^, ^6^ This result suggests that a significant number of Japanese patients may experience PPES when undergoing treatment with LEN, which may reduce the dose of LEN and limit therapeutic efficacy. However, the risk factors for PPES with LEN are not clear. Therefore, this study investigated the incidence of hypertension due to LEN and the relationships between PPES incidence and patient demographics by analyzing clinical laboratory data from patients with HCC receiving treatment according to LEN.

## 2 MATERIALS AND METHODS

### 2.1 Participants

Patients with HCC who received LEN between April 19, 2018, and September 30, 2020, were enrolled. The observation period was from 1 week before LEN administration to 1 month after LEN administration.

### 2.2 Data collection and PPES criteria

The following demographic data were collected: age, sex, height, body weight, body surface area, concomitant drug use, medical history, smoking history, and drinking history. The following clinical laboratory data were collected: systolic blood pressure, diastolic blood pressure, alanine aminotransferase, albumin (Alb), alkaline phosphatase (ALP), aspartate transaminase, C‐reactive protein, γ‐Glutamyl transpeptidase (γ‐GTP), lactate dehydrogenase, serum creatinine, total bilirubin (T‐Bil), urea nitrogen, uric acid, red blood cells, hemoglobin, hematocrit, platelets, basophils, eosinophils, lymphocytes, monocytes (MONO), neutrophils, LEN dose, and white blood cells. All data were collected from patient medical records. The albumin bilirubin score (ALBI) was also calculated from the Alb and T‐Bil values using following formula^8^:

$$\begin{array}{c} \text{ALBI}\;\text{score}=0.66\times \log_{10}\;\left(17.1\times T-\mathrm{Bil}\;\left[\mathrm{mg}/\mathrm{dL}\right]\right)\\ \;-\left(0.085\times 10\times \text{albumin}\;\left[g/\mathrm{dL}\right]\right) \end{array}$$

PPES was graded according to the Common Terminology Criteria for Adverse Events (CTCAE) version 5.0.^9^ A patient was considered to have PPES if an AE of Grade 1 or above was observed. Other AEs (hypothyroidism, hypertension, loss of appetite, diarrhea, and fatigue) were considered to have AE with a CTCAE Grade of 1 or above. In the absence of thyroid‐related tests, hypothyroidism was considered unassessable.

### 2.3 Statistical analyses

Continuous variables are expressed as medians [minimum and maximum], unless otherwise specified. Continuous variables were analyzed using the exact Mann–Whitney *U* test; categorical variables were analyzed using Fisher's exact test. The time‐dependent receiver operating characteristic (ROC) curves were estimated. The time trend of area under the curve (AUC) of the time dependent ROC curve was plotted, and cutoff values when AUC was maximum were set using the Youden index. Cox regression was used in univariate and multivariate analyses to calculate hazard ratios and 95% confidence intervals (CI). The variables with a *p* < 0.05 in the univariate analysis were included in the multivariate analyses and were selected using the forced selection method. The number of variables used in the multivariate analysis was adjusted to be less than the number of patients in the group with the lowest number of patients divided by 10. Multicollinearity was evaluated based on the variance inflation factor (VIF) with the cut‐off value set at 10. All variables used as predictors in the logistic regression were baseline values. All statistical analyses were conducted using R software (v.4.0.4).^10^ The “timeROC” package was used to estimate the time‐dependent ROC curve using inverse probability based on the censoring weighting method. Statistical significance was set at *p* < 0.05.

## 3 RESULTS

### 3.1 Demographic and clinical characteristics

**FIGURE 1:**
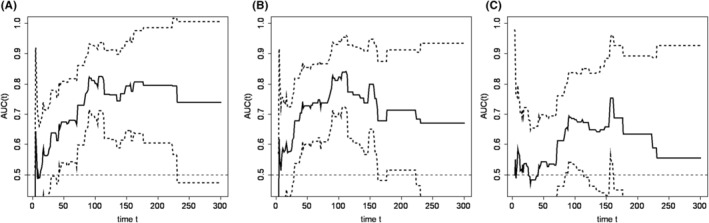
The time trend of area under the curve of the time dependent receiver operating characteristic curves for lenvatinib‐induced palmar–plantar erythrodysesthesia syndrome. (A) Alkaline phosphatase, (B) γ‐Glutamyl transpeptidase, (C) Monocytes. Solid line: Estimated area under the curve. Dotted line: 95% confidence interval for area under the curve. Vertical axis: AUC of time‐dependent ROC curve; Horizontal axis: number of days after start of LEN.

**FIGURE 2:**
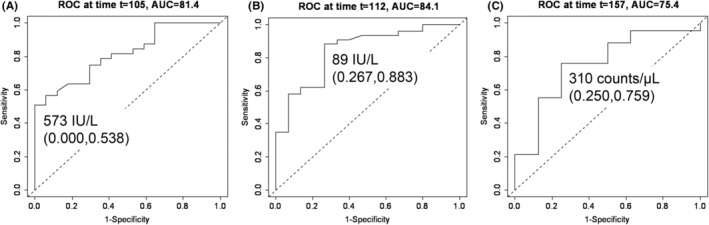
The receiver operating characteristic curve at the maximum area under the curve of the time‐dependent receiver operating characteristic curve. (A) Alkaline phosphatase at 105 days after start of LEN, (B) γ‐Glutamyl transpeptidase at 112 days after start of LEN, (C) Monocytes at 157 days after start of LEN. Vertical axis: sensitivity; horizontal axis: 1‐specificity.

Overall, 75 patients were enrolled in this study. PPES was found in 48.0% (36/75) of the patients. All patients received lifestyle guidance regarding standard preventive methods for PPES. Table 1 summarizes the baseline demographics of patients with and without LEN‐induced PPES. In those with LEN‐induced PPES, ALP, γ‐GTP, and MONO levels were significantly higher. Figure 1 shows the time trend of AUC of the time‐dependent ROC curves. ALP, γ‐GTP, and MONO had a 95% confidence interval of AUC greater than 0.5 within the time period studied. Figure 2 shows the ROC curve at the maximum AUC of the time‐dependent ROC curve. For ALP, γ‐GTP, and MONO, respectively, the AUC was greatest at 105 (AUC: 81.4), 112 (AUC: 84.1), and 157 days (AUC: 75.4) after LEN initiation. The cut‐off values of ALP, γ‐GTP, and MONO for patients with LEN‐induced PPES were estimated to be 573 U/L, 89 U/L, and 310 counts/μL, respectively.

**TABLE 1:** Baseline demographics.

Number of patients	No PPES (*n* = 39)	PPES (*n* = 36)	*p*
Sex (male/female)	32/7	27/9	0.575
Smoking history (+/‐)	30/9	28/8	1.000
Current smoking (+/‐)	9/30	15/21	0.136
Drinking history (+/‐)	18/21	24/12	0.381
Line of treatment (first/second or later)	28/11	31/5	0.164
CTCAE Grade for PPES (G1/G2/G3)	‐	19/11/6	‐

	**Median [minimum, maximum]**	**Median [minimum, maximum]**	
Age (years)	76 [49, 92]	72.5 [31, 87]	0.316
Height (cm)	161.8 [142.0, 177.7]	163.4 [147.4, 179.5]	0.589
BW (kg)	59.4 [37.7, 86.6]	62.4 [39.4, 80.9]	0.309
BSA (m^2^)	1.64 [1.24, 2.02]	1.66 [1.30, 1.96]	0.532
LEN initial dose (mg/day)	8 [4, 12]	12 [8, 12]	0.077
LEN dose per BW (mg/kg/day)	0.16 [0.05, 0.21]	0.17 [0.13, 0.20]	0.278
LEN dose per BSA (mg/m^2^/day)	5.76 [2.13, 7.33]	6.50 [4.70, 7.82]	0.131
Systolic BP (mmHg)	124 [100, 164]	123 [90, 160]	0.918
Diastolic BP (mmHg)	62 [42, 82]	63 [54, 80]	0.847
CRP (mg/dL)	0.40 [0.00, 8.50]	0.75 [0.00, 13.80]	0.105
ALB (g/dL)	3.5 [1.9, 4.7]	3.45 [2.3, 4.4]	0.633
ALBI	−2.16 [−3.27, −0.73]	−2.12 [−3.27, −1.34]	0.485
T‐BIL (mg/dL)	0.80 [0.20, 2.70]	0.90 [0.20, 1.80]	0.432
UN (mg/dL)	17 [9, 34]	15 [9, 45]	0.477
Cr (mg/dL)	0.79 [0.46, 5.04]	0.76 [0.39, 1.97]	0.667
eGFR (mL/min/1.73 m^2^)	72.8 [9.8, 128.1]	73.5 [26.4, 159.9]	0.691
UA (mg/dL)	5.3 [3.8, 8.2]	5.0 [1.9, 10.6]	0.255
AST (U/L)	42 [16, 135]	54 [18, 205]	0.189
ALT (U/L)	27 [8, 72]	29.5 [11, 155]	0.504
LDH (U/L)	235 [172, 508]	236 [134, 740]	0.948
ALP (U/L)	356 [180, 1998]	484.5 [204, 1386]	1.32 × 10^−3^
γ‐GTP (U/L)	111 [23, 455]	179.5 [17, 7033]	4.25 × 10^−3^
WBC (×10^3^/μL)	4.8 [2.1, 10.0]	5.5 [2.4, 15.8]	0.209
RBC (×10^6^/μL)	3.92 [2.44, 5.46]	3.81 [2.67, 6.48]	0.729
Hb (g/dL)	11.7 [8.6, 16.4]	12.6 [9.0, 17.2]	0.060
Ht (%)	34.9 [25.5, 48.6]	36.3 [26.1, 54.2]	0.119
PLT (×10^3^/μL)	146 [54, 451]	174 [50, 492]	0.496
BASO (/μL)	21.0 [0.0, 81.2]	20.0 [0.0, 134.0]	0.579
EOSINO (/μL)	151.2 [0.0, 2340.0]	88.0 [0.0, 275.0]	0.074
LYMPH (/μL)	1191.0 [221.4, 2838.0]	1290.0 [380.0, 2318.4]	0.322
MONO (/μL)	302.4 [123.9, 688.5]	429.4 [86.0, 1422.0]	0.013
NEUT (/μL)	2946.3 [1242.0, 8830.0]	3572.8 [1730.4, 8628.4]	0.391

Abbreviations: γ‐GTP, γ‐Glutamyl transpeptidase; ALB, albumin; ALBI, albumin bilirubin score; ALP, alkaline phosphatase; ALT, alanine aminotransferase; AST, aspartate aminotransferase; BASO, basophils; BP, blood pressure; BSA, body surface area; BW, body weight; Cr, serum creatinine; CTCAE, Common Terminology Criteria for Adverse Events; eGFR, estimated glomerular filtration rate; EOSINO, eosinophils; Hb, hemoglobin; Ht, hematocrit; LDH, lactate dehydrogenase; LEN, lenvatinib mesylate; LYMPH, lymphocytes; MONO, monocytes; NEUT, neutrophils; PLT, platelet; RBC, red blood cells; T‐BIL, total bilirubin; UA, uric acid; UN, urea nitrogen; WBC, white blood cells.

Table 2 shows the association between the presence of LEN‐induced PPES and the presence of other AEs. No significant associations with any other AEs were observed.

**TABLE 2:** Association of PPES with other adverse events.

Number of patients	No PPES (*n* = 39)	PPES (*n* = 36)	*p*
Hypothyroidism (±/unassessable)	25/4/10	29/3/4	0.225
Hypertension (+/‐)	27/12	29/7	0.298
Loss of appetite (+/‐)	15/24	18/18	0.381
Diarrhea (+/‐)	8/31	13/23	0.198
Fatigue (+/‐)	9/30	11/25	0.602

Abbreviation: PPES, palmar‐plantar erythrodysesthesia syndrome.

### 3.2 Cox regression analysis

**FIGURE 3:**
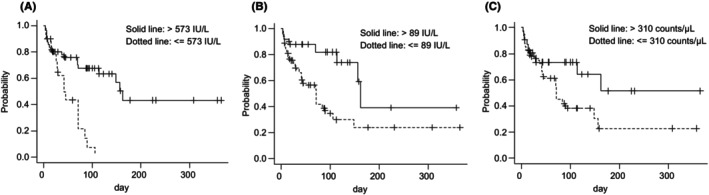
The Kaplan–Meier curve when classified by the cutoff values set in this study. (A) Alkaline phosphatase, (B) γ‐Glutamyl transpeptidase, (C) Monocytes.

Figure 3 shows the Kaplan–Meier curve when classified by the cut‐off values set in this study. Furthermore, Table 3 shows the Cox regression analysis results regarding LEN‐induced PPES predictors. Univariate Cox regression analysis was conducted using baseline demographic variables with *p* < 0.05. The crude hazard ratio of ALP (100 U/L increment), γ‐GTP (100 U/L increment), and MONO (100 counts/μL increment) for LEN‐induced PPES were 1.141, 1.057, and 1.177, respectively. Because ALP and γ‐GTP levels both reflect bile duct function, to avoid multicollinearity, we included only γ‐GTP, which had a lower P‐value in the univariate logistic regression model, in the regression equation.

**TABLE 3:** Results of Cox regression analysis of predictors of PPES.

Baseline demographic	Univariate analysis			Multivariate analysis			VIF
	Hazard ratio	95% CI	*p*	Hazard ratio	95% CI	*p*	
ALP (100 U/L increment)	1.141	1.054–1.236	0.001				
γ‐GTP (100 U/L increment)	1.057	1.026–1.088	2.2 × 10^−4^	1.051	1.012–1.335	0.001	1.004
MONO (100 counts/μL increment)	1.177	1.034–1.339	0.014	1.162	1.012–1.335	0.033	1.004

Abbreviation: γ‐GTP, γ‐Glutamyl transpeptidase; ALP, alkaline phosphatase; CI, confidence interval; MONO, monocytes; VIF, variance inflation factor.

The adjusted odds ratios of γ‐GTP (100 U/L increment) and MONO (100 counts/μL increment) for LEN‐induced PPES were 1.051 and 1.162, respectively. These two variables were significant explanatory variables even after adjusting for other variables. Because the VIFs were ≤10, we considered that there was no multicollinearity between these variables.

### 3.3 Prophylactic medication for PPES

**FIGURE 4:**
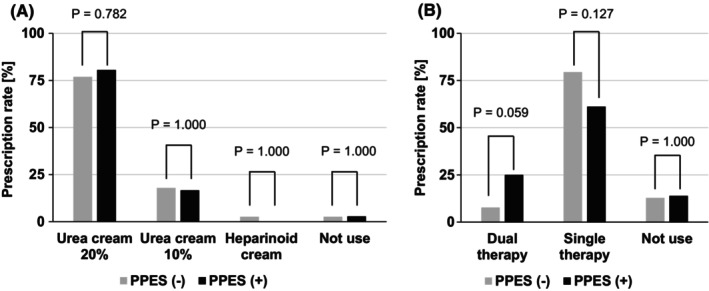
The prescription rate of prophylactic medication for lenvatinib‐induced palmar–plantar erythrodysesthesia syndrome. (A) Topical moisturizers, (B) Oral histamine H1 antagonist.

Figure 4 shows the prophylactic medications for LEN‐induced PPES. In this study, most patients received topical moisturizers (97.3%, 73/75) and oral histamine H1 antagonists (86.7%, 65/75). The topical moisturizer used was mostly urea cream (20%; 29/36 patients with PPES and 30/39 patients without PPES), followed by urea cream (10%; 6/36 patients with PPES and 7/39 patients without PPES). Heparinoid cream was used in only one patient without PPES. One patient in each group did not use a topical moisturizer. None of the statistical comparisons revealed significant intergroup differences. Oral histamine H1 antagonist therapy was the most commonly used monotherapy (22/36 patients with PPES and 31/39 patients without PPES), followed by combination therapy with two histamine H1 antagonists (9/36 patients with PPES and 3/39 patients without PPES).

## 4 DISCUSSION

In this study, the incidence of LEN‐induced PPES was 48.0%. In the REFLECT trials, LEN‐induced PPES has been reported in 51.9% (any Grade) in Japanese.^6^ The incidence of LEN‐induced PPES in the present study was similar to that in the REFLECT trial.

We identified high γ‐GTP and ALP levels as risk factors for PPES. ALP hydrolyses phosphate esters under alkaline conditions and is widely distributed in the body, including the liver, kidneys, osteoblasts, and small intestine, as well as the placenta. When inflammation or obstruction of the bile duct occurs, ALP present in epithelial cells flows into the blood, resulting in an increase in ALP level.^11^ Therefore, ALP is clinically used to evaluate liver function and, specifically, bile duct function. When LEN is administered to patients, it is metabolized in the liver and kidneys and excreted mainly in the bile.^12^ Accordingly, abnormalities in the bile duct system can affect LEN pharmacokinetics. A study evaluating liver function based on Child–Pugh classification and the effect of liver function on LEN pharmacokinetics reported no significant association between mild‐to‐moderate liver dysfunction and LEN pharmacokinetics.^13^ In a population analysis of LEN pharmacokinetics, liver function parameters, such as AST, ALT, and BIL, had no significant effect on pharmacokinetic parameters, whereas ALP and ALB did.^14^, ^15^ These reports suggest that elevated ALP has a negative effect on LEN clearance, with clearance becoming 0.855‐ to 0.888‐fold that when ALP levels exceed the upper reference limit. Although ALP has been used to assess liver function in previous studies, it also reflects bile duct function rather than liver function. In support of this, in the present study, we observed an increase in γ‐GTP, which reportedly reflects bile duct function, along with increased ALP level. To date, there are no reports on an association between γ‐GTP and LEN clearance; however, the present findings indicate that an increase in either ALP or γ‐GTP might reduce LEN clearance. In patients with high ALP or γ‐GTP, LEN clearance might decrease, and LEN blood levels might increase. Therefore, because high ALP or γ‐GTP levels are associated with an increase in blood LEN concentration, we suggest that the incidence of AEs increases as blood LEN concentration increases. However, LEN concentration and ALP isozymes were not evaluated in the present study. Because ALP is widely distributed in the body, ALP activity is usually calculated as a sum. When an increase in ALP is observed, it is possible to estimate the organ of origin by obtaining an isozyme fraction by electrophoresis. In the case of hepatobiliary disease, an increase in ALP1 or ALP2 is observed; thus, it is necessary to confirm whether bile duct dysfunction occurred in the patients included this study by isozyme analysis. Additionally, although blood levels were not measured in this study, factors that are strongly related to blood levels, such as ALP and γ‐GTP, and reportedly related to clearance might be risk factors for AEs. In contrast, no other AEs in this study were significantly associated with PPES. If all AEs are blood concentration‐dependent, this result is inconsistent with the explanations presented herein. However, few studies have examined the association or threshold between blood LEN concentration and each AE, and no AE has been clearly shown to be associated with blood LEN concentration. Although PPES may be most strongly associated with blood LEN concentration among LEN induced AEs, information on blood LEN concentration is lacking in this study. Therefore, blood levels of ALP or γ‐GTP and LEN concentration and their correlations remain to be examined in detail in future work.

Moreover, in the present study, ALBI was not significantly associated with PPES expression. A previous study reported that high grade ALBI is associated with discontinuation due to AEs.^16^ However, since ALBI was not considered as a risk factor for each AE in that study, we believe that AEs other than PPES may be associated with ALBI.

In addition, PPES expression has been reported to prolong overall survival and progression‐free survival.^16^, ^17^ However, our study did not investigate efficacy measures such as overall survival, so the relationship between side effects such as PPES and efficacy is unclear. However, if our hypothesis that PPES is associated with decreased LEN clearance is correct, an association between efficacy and blood concentration of LEN may exist. Based on these considerations, we believe that starting LEN at an underdose with an emphasis on prevention of PPES is not recommended to ensure the efficacy of LEN and that monitoring using objective indicators such as blood levels of LEN should be verified. Furthermore, appropriate dose reduction methods should be included when considering the continuation of LEN treatment. In recent years, modified administration methods such as taking the drug for 5 days with 2‐day intervals (weekends‐off method) is reported to decrease discontinuations of LEN treatment.^18^, ^19^ However, information concerning the pharmacokinetics of LEN is unknown in these studies. Since the weekends‐off method was not incorporated in this study, we believe that further research is needed.

We identified high MONO levels as risk factors for PPES, even after adjusting for other variables in the multivariate logistic regression model. To the best of our knowledge, there are no previous reports on the association between leukocyte fractions and LEN‐induced PPES. There exist various MONO subpopulations, which have various physiological effects. The classical population (CD14^++^, CD16^−^) is involved in angiogenesis, proliferation, and inflammation, and the non‐classical population (CD14^+^, CD16^++^) is involved in apoptosis, antiproliferative regulation, and cytoskeletal stability.^20^ Therefore, additional information can be obtained by analyzing MONO subpopulations. The mechanism of PPES has not yet been clarified; however, several mechanisms are being considered, including the following. (1) Anti‐VEGF drugs might reduce the ability of endothelial cells to regenerate due to trauma.^21^, ^22^ (2) PPES is caused by inhibiting KIT, a PDGF receptor present in the ductal epithelium of eccrine sweat glands^22^, ^23^, ^24^ (3) Inhibition of KIT might by toxic to keratinocytes.^22^, ^25^ (4) RET and Flt‐3 are related to PPES incidence.^22^, ^26^ Because MONO is associated with inflammation, patients with PPES might have experienced inflammation, such as mild trauma that induced PPES.

The prescription rates of moisturizers and antihistamines as prophylactic agents did not differ in the absence or presence of PPES. Moisturizers are used to protect against PPES during sorafenib treatment and currently represent the most common prophylactic agents,^27^ whereas antihistamines are less commonly used. These prophylactic agents (e.g., moisturizers and antihistamines) showed no clear preventive effect in the present study. In particular, a possible reason for the lack of efficacy regarding moisturizers was the very high rate of prescription of moisturizers (97.3%), which resulted in inadequate comparisons with patients who did not use moisturizers. In future work, it will be necessary to consider appropriate preventive drugs, as it is important to establish PPES management in order to continue LEN treatment at appropriate doses.

This study had several limitations. First, unmeasured confounding factors could not be adjusted. Therefore, it is unclear how the study results are influenced by other confounding factors. Second, the number of cases was relatively small (*n* = 75). Validation through larger prospective studies is desired. Third, our data are a survey of risk factors related to PPES for any grade. Therefore, the results may differ when restricted to higher grades, for example. These analyses could not be performed based on the small number of cases in our study. Fourth, this study hypothesizes that inflammatory status prior to LEN use is a risk factor for PPES based on the association between PPES and MONO, but information on its association with other inflammatory responses and inflammation‐related side effects is lacking. We believe that further studies are needed to confirm this hypothesis. Finally, our research did not assess the effects of LEN. Therefore, further research is needed.

In conclusion, high γ‐GTP and high MONO were identified as risk factors for LEN‐induced PPES; appropriate assessment of PPES risk and early intervention on PPES prevention for high‐risk patients may result in continued treatment with LEN.

## AUTHOR CONTRIBUTIONS

**Shusuke Uekusa:** Data curation (lead); formal analysis (lead); investigation (supporting); resources (lead); software (equal); validation (equal); visualization (equal); writing – original draft (equal); writing – review and editing (lead). **Maho Nemoto:** Data curation (supporting); formal analysis (supporting); investigation (supporting); resources (lead); writing – review and editing (supporting). **Yuki Hanai:** Data curation (supporting); formal analysis (supporting); investigation (supporting); resources (supporting); writing – review and editing (supporting). **Misaki Nakashin:** Data curation (supporting); formal analysis (supporting); resources (supporting); writing – review and editing (supporting). **Sachiko Yanagino:** Resources (supporting). **Yoshiki Arita:** Resources (supporting). **Takahiro Matsumoto:** Resources (supporting). **Noritaka Wakui:** Conceptualization (supporting); methodology (supporting); project administration (supporting); resources (supporting); writing – review and editing (supporting). **Hidenari Nagai:** Conceptualization (lead); methodology (supporting); project administration (supporting); resources (supporting); writing – review and editing (supporting). **Koji Higai:** Conceptualization (lead); methodology (lead); project administration (supporting); writing – review and editing (supporting). **Kazuhiro Matsuo:** Conceptualization (lead); methodology (lead); project administration (lead); writing – review and editing (lead).

## FUNDING INFORMATION

There are no funding sources for this study.

## CONFLICT OF INTEREST STATEMENT

Shusuke Uekusa has no conflict of interest; Maho Nemoto has no conflict of interest: Yuki Hanai has no conflict of interest; Misaki Nakashin has no conflict of interest; Sachiko Yanagino has no conflict of interest; Yoshiki Arita has no conflict of interest; Takahiro Matsumoto has no conflict of interest; Noritaka Wakui has no conflict of interest; Hidenari Nagai received research grant from Eisai, Otsuka Pharma, and AbbVie; Koji Higai has no conflict of interest; Kazuhiro Matsuo has no conflict of interest.

## ETHICS STATEMENT

This was a single‐centre, retrospective study. The study protocol was approved by the Ethics Committee of Toho University Omori Medical Center (approval number: M19249). The requirement for informed consent was waived by the review board because this study was retrospective and observational, and all data were fully de‐identified. We confirm that we have read the journal's guidelines on issues involved in ethical publication and affirm that this report is consistent with these guidelines.

## Data Availability

The data are not publicly available for ethical reasons. Further inquiries can be directed to the corresponding authors.
